# DDmap: a MATLAB package for the double digest problem using multiple genetic operators

**DOI:** 10.1186/s12859-019-2862-x

**Published:** 2019-06-18

**Authors:** Licheng Wang, Jingwen Suo, Yun Pan, Lixiang Li

**Affiliations:** 1 State Key Laboratory of Networking and Switching Technology, Beijing University of Posts and Telecommunications, 10 West Tucheng Road, Haidian District, Beijing, 100876 China; 2 School of Computer Science, Communication University of China, 1 East of Dingfuzhuang Street, Chaoyang District, Beijing, 100024 China

## Abstract

**Background:**

In computational biology, the physical mapping of DNA is a key problem. We know that the double digest problem (DDP) is NP-complete. Many algorithms have been proposed for solving the DDP, although it is still far from being resolved.

**Results:**

We present DDmap, an open-source MATLAB package for solving the DDP, based on a newly designed genetic algorithm that combines six genetic operators in searching for optimal solutions. We test the performance of DDmap by using a typical DDP dataset, and we depict exact solutions to these DDP instances in an explicit manner. In addition, we propose an approximate method for solving some hard DDP scenarios via a scaling-rounding-adjusting process.

**Conclusions:**

For typical DDP test instances, DDmap finds exact solutions within approximately 1 s. Based on our simulations on 1000 random DDP instances by using DDmap, we find that the maximum length of the combining fragments has observable effects towards genetic algorithms for solving the DDP problem. In addition, a Maple source code for illustrating DDP solutions as nested pie charts is also included.

## Background

The physical mapping of DNA is a key problem in computational biology [5]. A large DNA molecule is a long string composed of four nucleotides, A, C, G and T. To understand the structure of DNA molecules, it is of interest to determine the occurrences of short substrings, such as GAATTC, on the DNA. Double digest experiments (DDE for short) are a standard approach for constructing physical DNA maps [2]. Given two restriction enzymes, denoted by $\mathfrak{A}$ and $\mathfrak{B}$, this approach cuts a target DNA sequence by using only enzyme $\mathfrak{A}$, only enzyme $\mathfrak{B}$, and both enzymes simultaneously, in three separate and parallel experiments [5]. As a result, we obtain three multisets of short DNA fragments. However, due to certain experimental limitations, only the length information (i.e., The number of nucleotides) of these short fragments can be measured with certain accuracy using certain mature biological techniques, such as gel electrophoresis. The objective of the double digest problem (DDP) is to reconstruct the original ordering of the fragments in the target DNA molecule.

Since the first successful reconstruction of restriction site mapping in the earlier 1970s [7, 11], the DDP problem has become an intensively studied issue that covers a variety of disciplines [6, 9]. Although the major concerns come from the community of bioinformation, the challenges related to this problem have also attracted attention from the artificial intelligence, algorithmic complexity, and optimization communities. We now know that DDP is strongly NP-complete [1, 2], and many algorithms have been proposed for solving the DDP problem [3–6, 8–10, 12–15]. However, the DDP problem is still far from being resolved. All of the algorithms developed to address this problem have encountered significant difficulties as the number of restriction sites increases. Moreover, even for different DDP instances with the same size, the hardness for finding an exact solution might vary remarkably.

The main motivation of this work comes from three considerations: First, almost all existing formulations of the DDP problem use multiset as the basic data structure, while we find that it is even easier to model the DDP problem by using vectors. Second, some recently proposed genetic algorithms [3, 13] for addressing the DDP problem should be improved. Third, it is of interest to develop an open-source package for studying the DDP problem by using easily accessible engineering computation platforms, such as MATLAB.

Our main contributions are summarized as follows:

- A vector-based formulation of the DDP problem is presented and illustrated step-by-step.
- A novel genetic algorithm for solving the DDP problem is proposed by combining six genetic operators, and a MATLAB package, DDmap, is implemented by integrating the proposed genetic algorithm and other necessary supporting and testing widgets. Then, by using DDmap, exact solutions for typical DDP test instances [13] are explicitly derived and depicted. (See the right column of Table 1.)
- A relation between the hardness of certain DDP instances and the maximum length of double digest sequences is revealed based on our simulations of 1000 random DDP instances. Meanwhile, an approximate approach for typical hard DDP instances is conceived based on this relation.

**Table 1:**
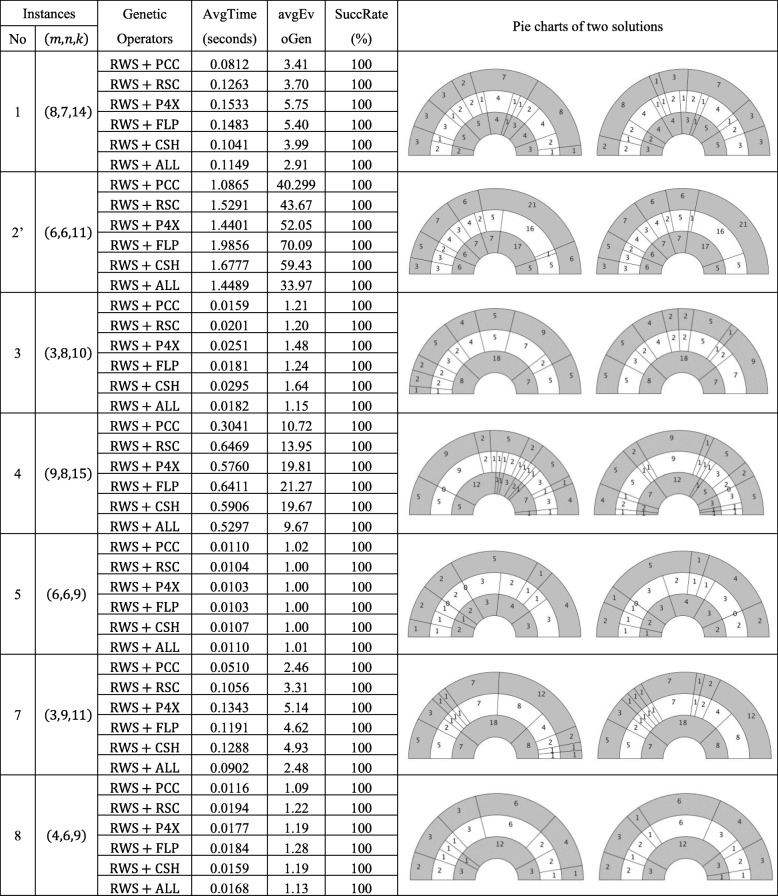
Main results: separated and integrated effects of all six genetic operators. Instance 1,3,4,5,7,8 come from [13], instance 2' is derived by using a scaling-rounding-adjusting process towards instance 2, *m*, *n*, *k* are the lengths of the input fragments A, B and C, respectively. There are six genetic operators, RWS is selection operator defined as the well-known roulette wheel algorithm. PCC and RSC are crossing operators, PCC is the combination of two permutations, RSC is Referencing Sorting Crossing, P4X, FLP, CSH are mutation operators, P4X is a four-point mutating, FLP defined as the flipping of the given fragment. CSH defined as the cyclic shifting of the given fragment. The average running time, average evolution generations and success rate are listed in the table. At the right of the table, we draw pie charts of DDmap’s two solutions.

## Results

To test the utility of DDmap, eight DDP instances, referred to as INS_*j*_(*j* = 1⋯8), are taken from [13]. They are shown in the following Table 2:

**Table 2:**
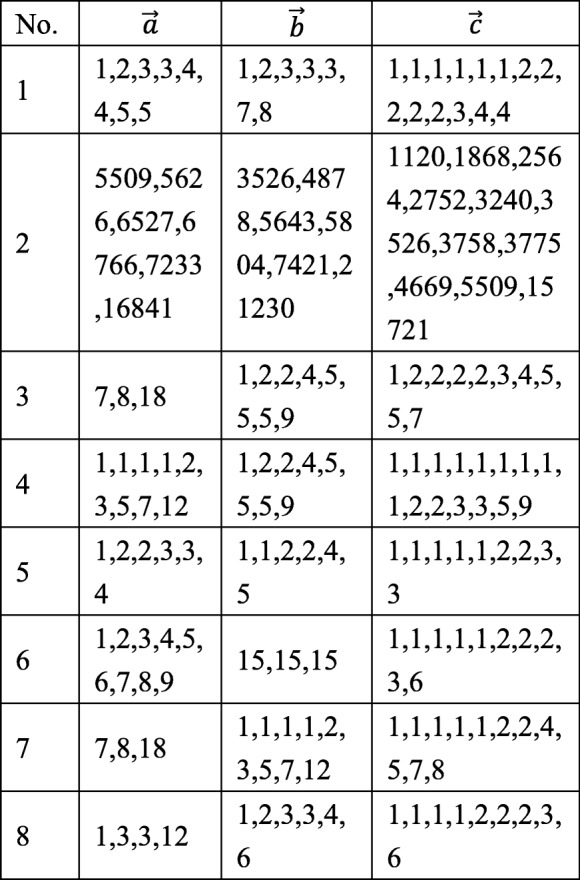
Test instances from [13]. Suppose giving two restriction enzymes, denoted by $\mathfrak{A}$ and $\mathfrak{B}$, $\overrightarrow{a},\overrightarrow{b},\overrightarrow{c}$ are the multisets of short DNA fragments by cuts a target DNA sequence by using enzyme $\mathfrak{A}$ only, enzyme $\mathfrak{B}$ only, and both enzymes simultaneously.

**Fig. 1:**
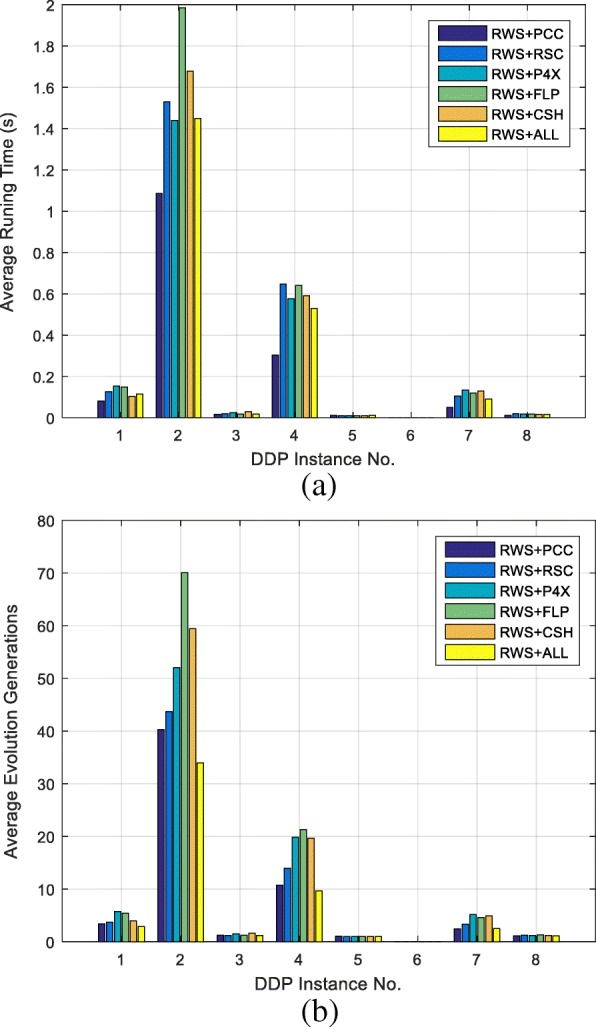
Main results: separated and integrated effects of all six genetic operators. **a** is the average running time. **b** is the average evolution generations. DDmap has six genetic operators, for each instance, 100 trails were run by using DDmap with respect to each combination of six genetic operators. Then draw bar charts of the average running time. INS_6_ doesn’t have data because it is invalid

First, the integrated effects of the six aforementioned genetic operators of DDmap are verified. For the instances INS_1, 3, 4, 5, 7, 8_, DDmap performs considerably well, and the related results are collected in Table 1. For *each* instance, 100 trails were run using DDmap with respect to *each* combination of six genetic operators. Then, the average running time, the average evolution generation and the success rate of finding exact DDP solutions are counted. Two different exact solutions for the instances *INS*_1, 3, 4, 5, 7, 8_ are also depicted in the right column of Table 1. In addition, the average running time and the average evolution generations of finding exact DDP solutions are depicted in Fig. 1. From Table 1 and Fig. 1. We can see that the genetic operators combination of RWS + PCC performs best in running time, RWS + ALL performs best in evolving generation, while other combinations of different genetic operators perform similarly and equally effective. Moreover, the tendency of running time curve and evolving generation curve are very similar.

However, we find that DDmap performs very poorly for INS_2_ and INS_6_. Upon further examination, we find that INS_6_, is *invalid*, Simple calculation shows that as for INS_6_, we have

$$ 45=\sum \left(\overrightarrow{a}\right)=\sum \left(\overrightarrow{b}\right)\ne \sum \left(\overrightarrow{c}\right)=19 $$

because it violates the restriction condition of (5) (See Definition 1).

**Fig. 2:**
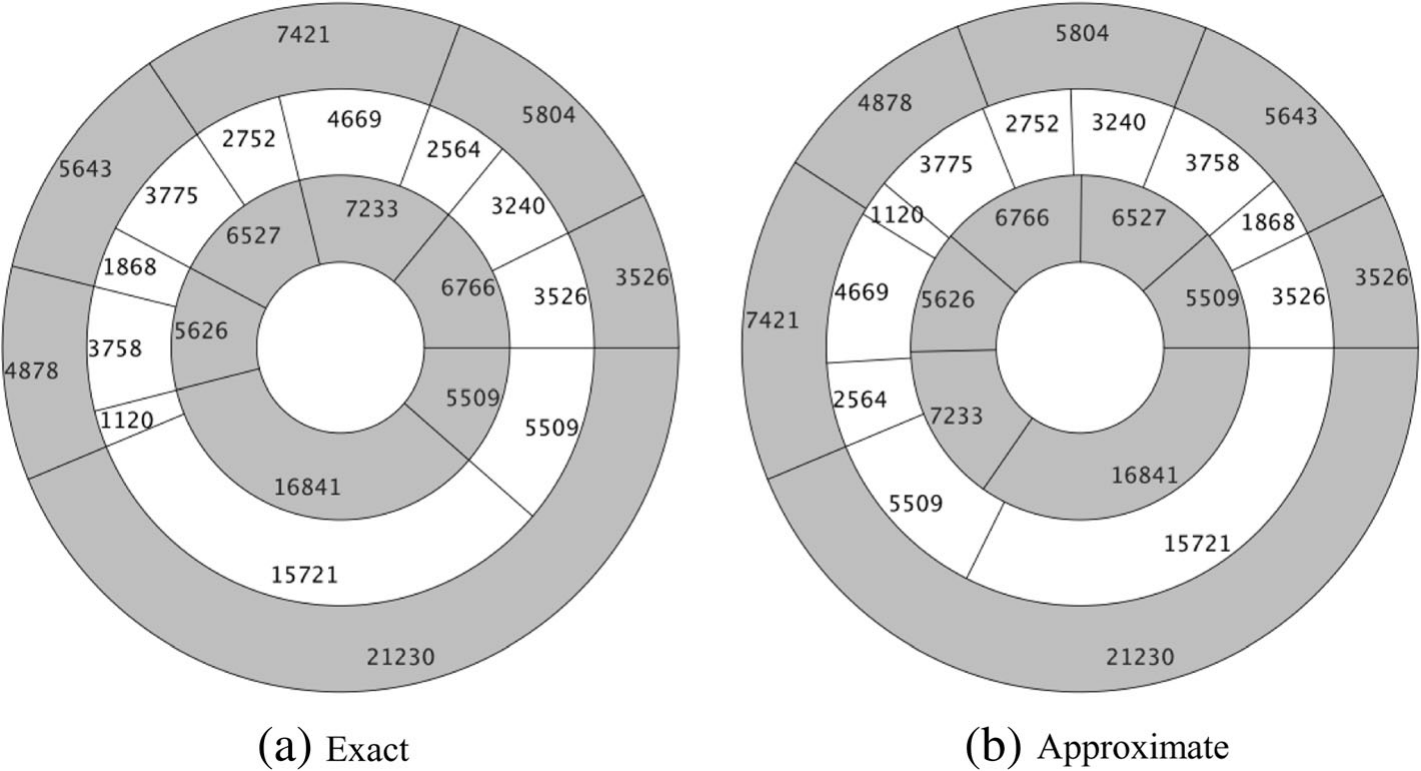
Effects of scaling-rounding-adjusting method. **a** is an exact solution of INS_2_. **b** is an approximate solution of INS_2_, derived by using the scaling-rounding-adjusting process towards INS_2_

For INS_2_, we run DDmap 100 trails and successfully obtain exact solutions of INS_2_ in 67 trails. But the average running time and evolution generations for reaching the exact solution of INS_2_ are 122 s and 3828, respectively, i.e., approximately 1000 times slower than the results of other test instances (see Table 1). Furthermore, we find that these 67 solutions are essentially the same: One solution is depicted in Fig. 2(a), and another solution is just to read out the sequences A, B and C of Fig. 2(a) in an reverse order. It seems that the solution to INS_2_ ’s solutions are very sparse, and thus, DDmap faces the difficulty of escaping from so many local optima.

We deal with the INS_2_ by using the scaling-rounding-adjusting approach. As expected, DDmap can find solutions towards INS_2′_ very efficiently. For each combination of six genetic operators, we run DDmap towards INS_2′_ 100 trials. The average running time is no more than 2 s, the evolution generation is no more than 80, and the success rate for finding exact DDP solutions is always 100%. The results are already contained in Table 1 and Fig. 1. Now, we directly take some INS_2_, ’s solution, (*μ*, *ν*) ∈ *S*_*m*_ × *S*_*n*_, as an approximate solution of INS_2_. The resulted double digest pie charts are depicted in Fig. 2(b). Compared to the exact solution given in Fig. 2(a), we think this kind of approximation is an interesting result in the sense that the relative error, defined as the proportion of total length of gaps between two miss-aligned fragments, is merely 4.8%, calculated by

$$ \frac{115+17+256+171+117+188+280+1120}{48502}=0.0487. $$

**Fig. 3:**
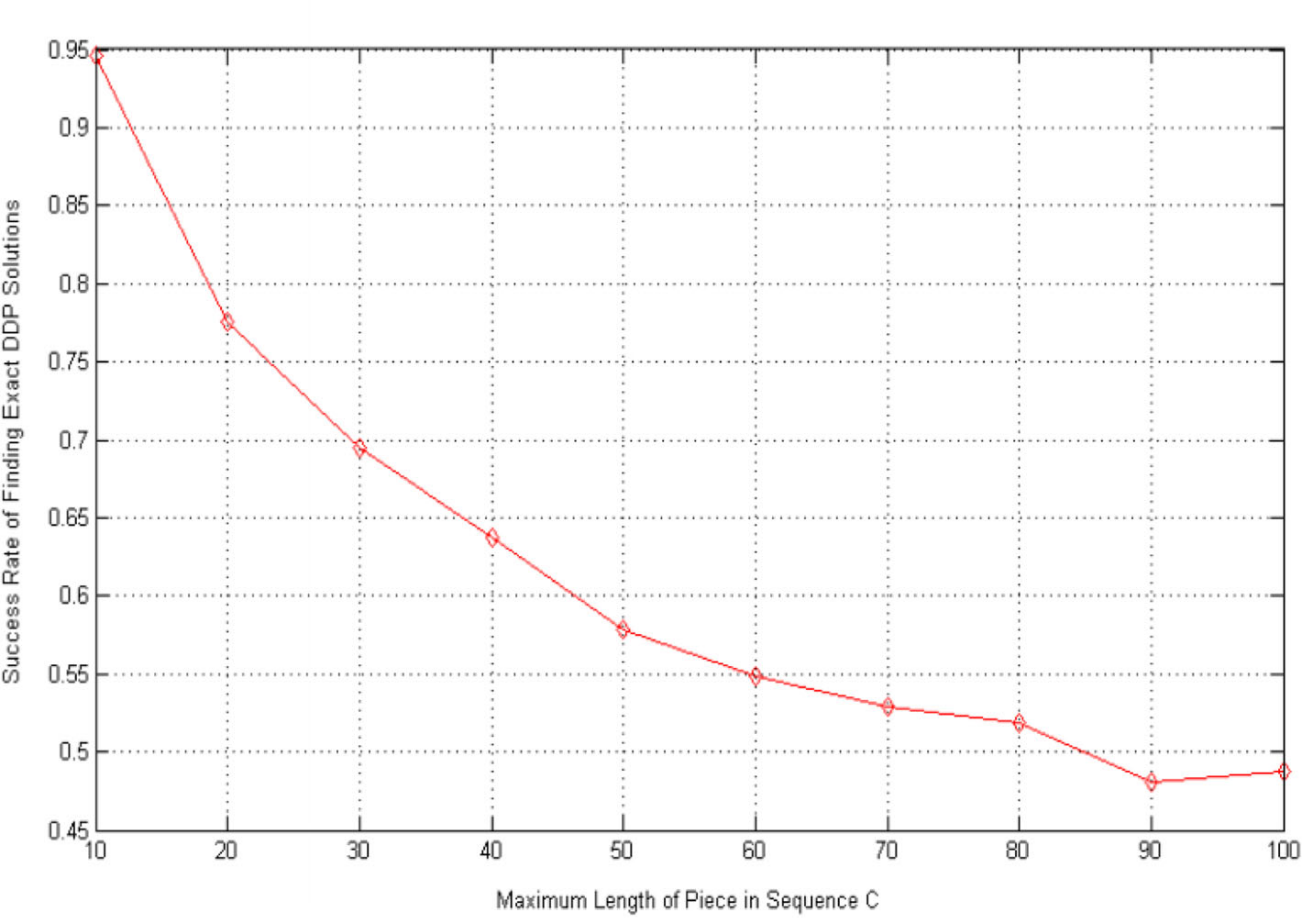
Success Rate vs. Maximum Length of Piece in C. DDmap’s performance is tightly related to the maximum length of piece in C, we generated a series of random double digest instances with the maximum length of C ranging from 10 to 100, then test the DDmap’s success rate, the line of success rate changing with the maximum length of C is shown in Fig. 3

Next, via a number of simulations, we find that DDmap’s performance is tightly related to the maximum length of a piece in the sequence of C, denoted by *ρ*_*C*_ = max *c*_*i*_. This is reasonable considering that for a fixed length of sequence C, denoted by *L*_*C*_ = |*C*|, the smaller *ρ*_*C*_ is, the denser the solutions, and thus, the easier for genetic algorithms, such as DDmap, to meet an exact solution during the evolution process. Based on our simulations towards 1000 random DDP instances with different *ρ*_*C*_, the relationship between the success rate of finding exact DDP solutions with respect to *ρ*_*C*_ is depicted in Fig. 3.

## Discussion

- ♦ Cases of k ≠ m + n − 1

Note that in both INS_4_ and INS_5_, the given two enzymes cut the target DNA molecule at some of the same sites and lead to the case where k ≠ m + n − 1. At the beginning, DDmap performs very poorly on INS_4_ and INS_5_. The performance of DDmap on INS_4_ and INS_5_ improves remarkably after we adopt the following simple preprocessing strategy:

• If k < m + n − 1, then introduce δ = (m + n − 1) − k fragments with length 0 into.

the sequence $$ \overrightarrow{c} $$;

• Otherwise, if k > m + n − 1, then introduce δ = k − (m + n − 1) fragments with length 0.

into the shorter sequence among $\overrightarrow{a}$ and $\overrightarrow{b}$;

• Otherwise, do nothing.

**Fig. 4:**
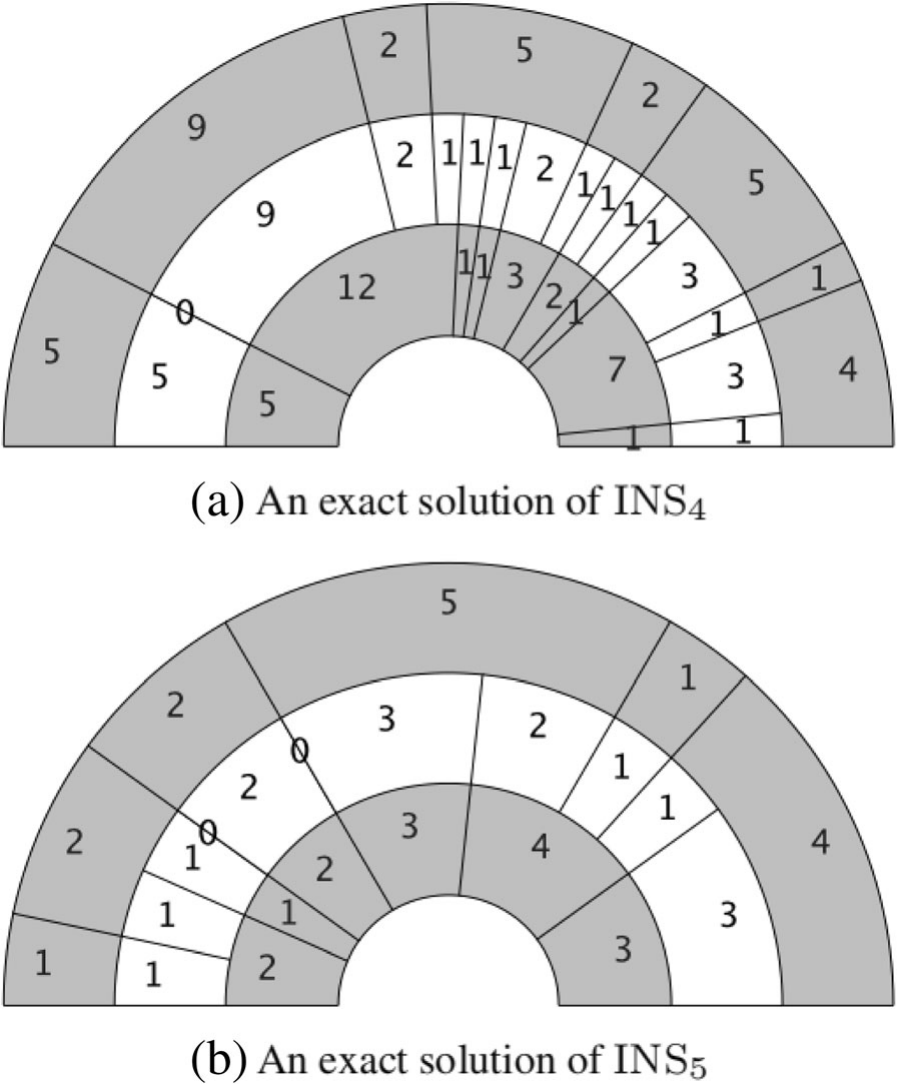
Appearance of 0-length fragments *m*, *n*, *k* are the length of the input instance A B and C, when k ≠ m + n − 1, We introduce some 0-length fragments into the sequence, (**a**) shows that a 0-length fragment in sequence $\overrightarrow{c}$ of INS_4_ appears at the fifteenth site, (**b**) shows that two 0-length fragments in sequence $\overrightarrow{c}$ of INS_5_ appear at the sixth and the eighth sites, respectively

An interesting observation is that the newly introduced 0-length fragments will explicitly appear in the pie charts of exact DDP solutions. For instance, Fig. 4(a) shows that a 0-length fragment in sequence $\overrightarrow{c}$ of INS_4_ appears at the fifteenth site, while Fig. 4(b) shows that two 0-length fragments in sequence $\overrightarrow{c}$ of INS_5_ appear at the sixth and eighth sites, respectively.

Here, we follow the convention of reading a pie chart from 0^°^ to 180^°^ or 360^°^.

- ♦ Comparison

**Fig. 5:**
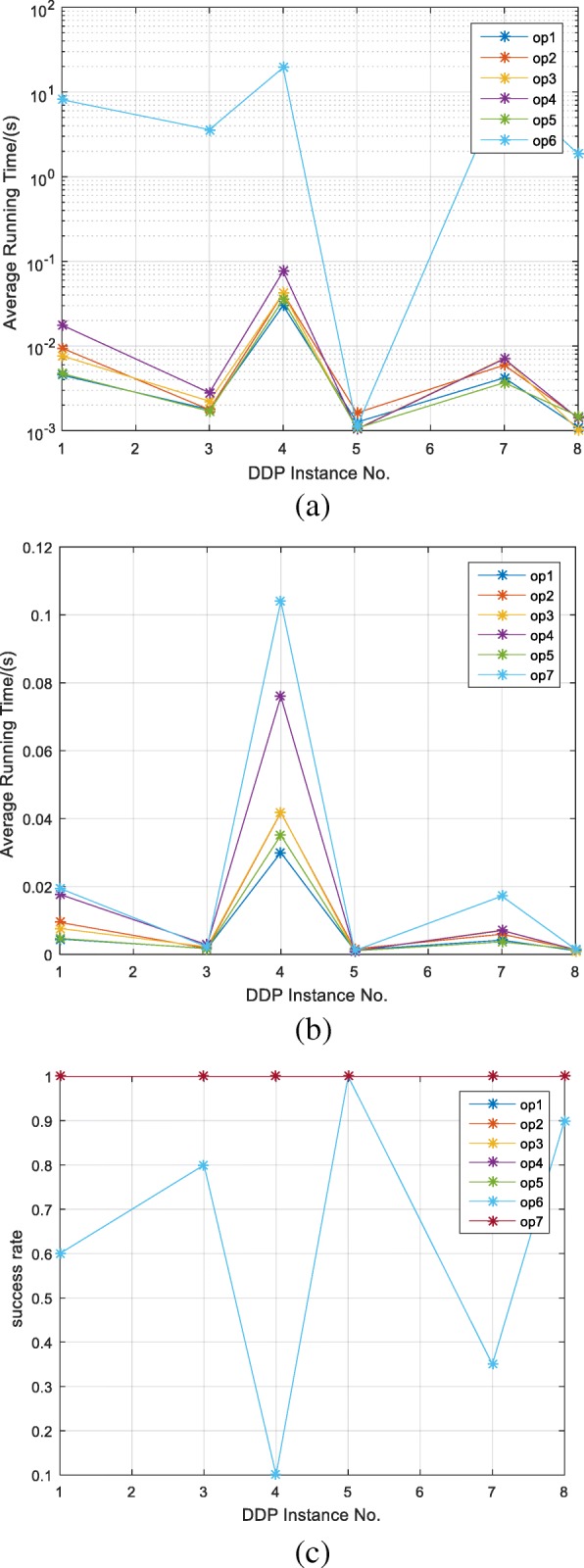
Comparison of DDmap and algorithm in [3, 13]. Operators 1–5 are the crossover and mutation operators in DDmap, op6 is the mutation operator in [13] and op7 is the crossover operator in [3]. Each instance is run 100 times by using op1–7 respectively. **a** is a logarithmic coordinate system figure, we can see the average running time comparison between DDmap and the algorithm in [13] in (**a**). **b** is the average running time comparison between DDmap and the algorithm in [3]. **c** is the success rate comparison between DDmap and the algorithm in [3, 13]

Figure 5(a) and (b) are the comparison of the average running time between DDmap and the algorithm in 2005 [13] and 2012 [3]. Operator 1–5 are the crossover and mutation operator in DDmap. Because the crossover operator in [13] is the same as our operator 2 and the two mutation operators in [3] are similar to our operators op4 and op5, so we only implement the mutation operator op6 in [13] and crossover operator op7 in [3]. Eight instances are from the paper [13]. In the comparison experiment, each instance is run 100 times for operators op1–7 respectively, and then we got the average running time and the success rate of finding the exact DDP solution.

Through the experimental data, we found the data of op6 is much larger than that of the other six operators, the data of the other six operators will be neglected in the rectangular coordinate system, so we choose the logarithmic coordinate system. Figure 5(a) is the comparison between DDmap and the algorithm in 2005 [13], the blue line is the average running time of op6, it is higher than the other six lines, our algorithm has a significant time advantage over the [3]‘s algorithm. As can be seen from Fig. 5(b), the six lines have little difference, however, the op7’s line is always at the top, so our algorithm has a slight advantage over that of [3].

The comparison of success rate is shown in Fig. 5(c). The success rate of operators 1, 2, 3, 4, 5, 7 is 100%, they are all effective for these instances. Operator 6 runs very irregularly and the results are not very good.

**Fig. 6:**
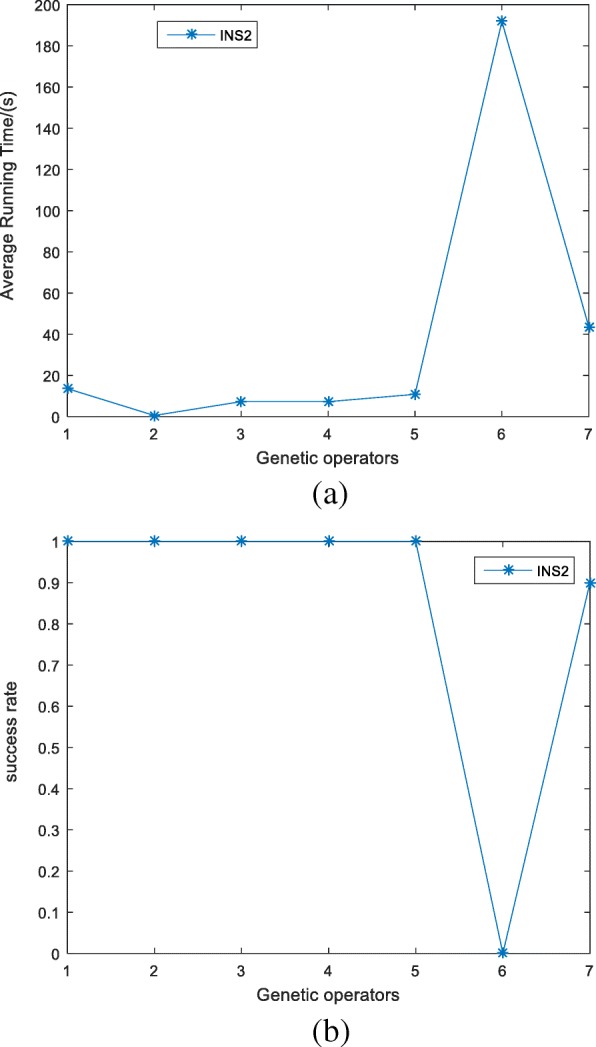
Comparison of DDmap and algorithm in [3, 13] under the condition of INS_2_. The maximum evolution generation is set to 100,000, running each operator 10 times, (**a**) is the average running time of each operator. **b** is the success rate of each operator

Instance 2 and 6 does not appear in Fig. 5. In fact, INS_6_ is invalid. As aforementioned, INS_2_ is very complex, so we analyze it separately. To reset the maximum evolution generation as large as 100,000, running each operator 10 times towards INS_2_, the average running time and the success rate is shown in Fig. 6(a) and (b), respectively. We can see that the running time of op6 is about 10 times longer than other operators, while the running time of op7 is about twice longer than our operators op1–5. The success rates of our five operators are all 100%, however, op7’s success rate is 90%, but op6 does not produce the exact DDP solution.

**Fig. 7:**
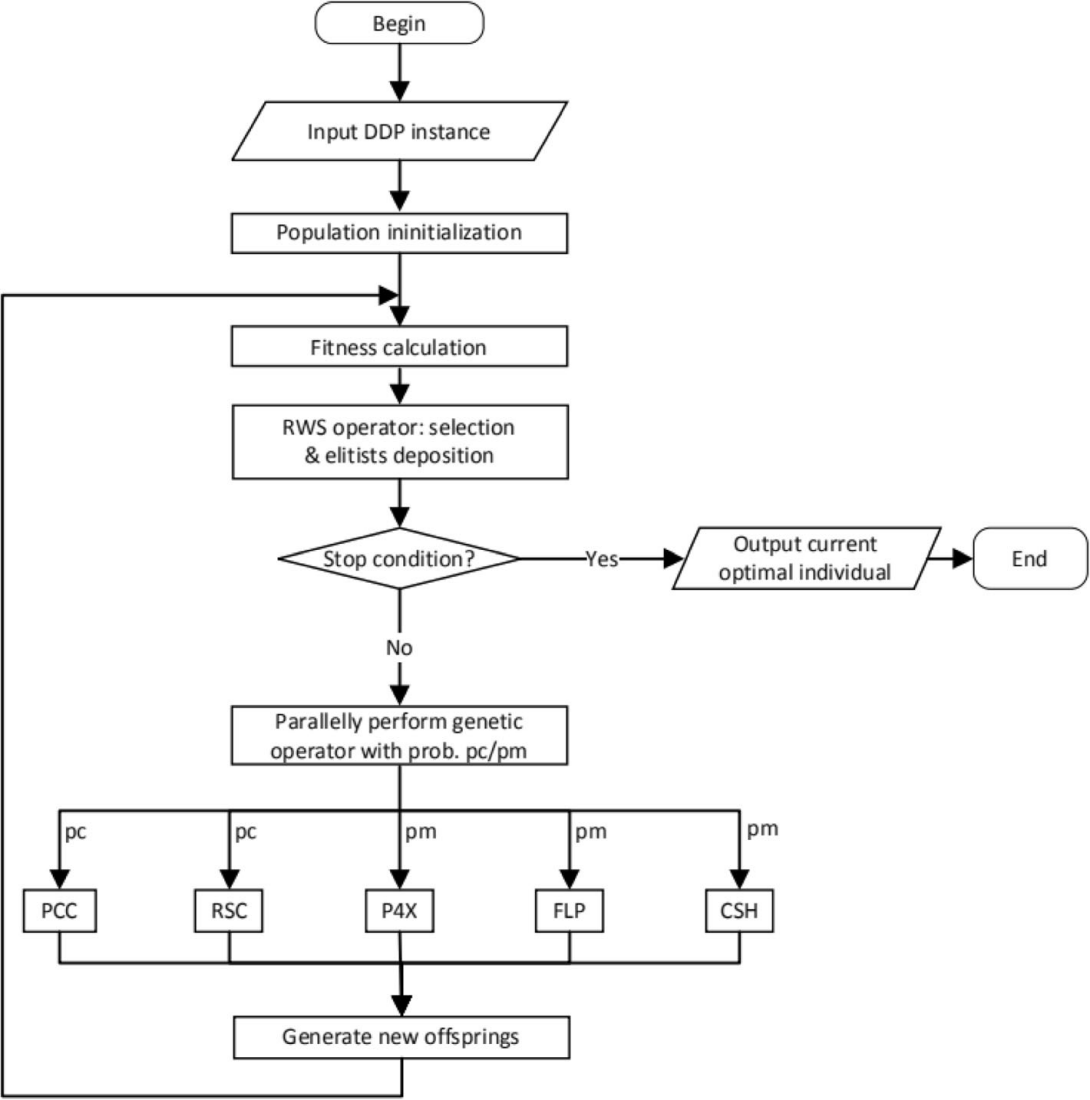
Flowchart of main GA algorithm of DDmap. The input DDP instance includes the instances in [13] and random instances, after calculating the fitness value, if not satisfied the stop condition, the crossover and mutation operators will be performed probabilistically, then generate new offsprings and recalculate the fitness values

In conclusion, DDmap is much better than the algorithm in [13] and it is slightly better than [3]’s algorithm.

## Conclusions

An open-source MATLAB package DDmap based on a newly designed genetic algorithm that combines six genetic operators is designed for solving the double digest problem. This algorithm finds exact solutions within approximately 1 s for typical DDP test instances. For some hard DDP instances, DDmap performs very well via a scaling-rounding-adjusting process. The experimental results of our algorithm confirm its efficiency.

## Methods

### Problem formulation

Let *S*_*m*_ denote the symmetric group on m indices {1, 2, ⋯, *m*}. Then, for a given permutation π ∈ *S*_*m*_ and a given vector $\overrightarrow{a}=\left({a}_1,\cdots, {a}_m\right)$, the action of π on $\overrightarrow{\mathrm{a}}$ derives a vector ${\overrightarrow{a}}^{\pi }=\left({a}_{\pi (i)},\cdots, {a}_{\pi (m)}\right)$, reassembling of the order of entries of $\overrightarrow{\mathrm{a}}$ according to π. Further, let us define the *accumulative sum vector* of $\overrightarrow{\mathrm{a}}$, denoted by $\mathrm{AS}\left(\overrightarrow{\mathrm{a}}\right)$, and the *step difference vector* of $\overrightarrow{\mathrm{a}}$, denoted by $\mathrm{AS}\left(\overrightarrow{\mathrm{a}}\right)$, as follows:

1 $$ \mathrm{AS}\left(\overrightarrow{\mathrm{a}}\right)=\left(\Sigma \left(\overrightarrow{a},1\right),\cdots, \Sigma \left(\overrightarrow{a},\mathrm{m}\right)\right) $$

and

2 $$ \mathrm{SD}\left(\overrightarrow{\mathrm{a}}\right)=\left(\sum \left(\overrightarrow{a},1\right),\sum \left(\overrightarrow{a},2\right)-\sum \left(\overrightarrow{a},1\right),\cdots, \sum \left(\overrightarrow{a},\mathrm{m}\right)-\sum \left(\overrightarrow{a},\mathrm{m}-1\right)\right) $$

where $\Sigma \left(\overrightarrow{a},\mathrm{j}\right)={\sum}_{i=1}^j{a}_i\left(j=1,\cdots, m\right)$ indicates the partial sum of $\overrightarrow{\mathrm{a}}$.

Now, the double digest problem (DDP) can be formulated by the following steps:

- Given two vectors $\overrightarrow{a}=\left({a}_1,\cdots, {a}_m\right)$ and $\overrightarrow{b}=\left({b}_1,\cdots, {b}_n\right)$ with the restriction $\Sigma \left(\overrightarrow{a},\mathrm{m}\right)=\Sigma \left(\overrightarrow{b},\mathrm{n}\right)$, we define the combining sequence of $\overrightarrow{\mathrm{a}}$ and $\overrightarrow{\mathrm{b}}$, denoted by $\coprod \left(\overrightarrow{a},\overrightarrow{b}\right)$, as the concatenation of vectors $\mathrm{AS}\left(\overrightarrow{\mathrm{a}}\right)$ and $\mathrm{AS}\left(\overrightarrow{\mathrm{b}}\right)$ and removing the tail entry. That is,

3 $$ \coprod \left(\overrightarrow{a},\overrightarrow{b}\right)=\left(\mathrm{AS}{\left(\overrightarrow{\mathrm{a}}\right)}_1,\cdots, \mathrm{AS}{\left(\overrightarrow{\mathrm{a}}\right)}_m,\mathrm{AS}{\left(\overrightarrow{\mathrm{b}}\right)}_1,\cdots, \mathrm{AS}{\left(\overrightarrow{\mathrm{b}}\right)}_{n-1}\right) $$

- The sequence $\coprod \left(\overrightarrow{a},\overrightarrow{b}\right)$ can be reassembled to obtain a new sequence according to the nondecreasing order, denoted by $\hat{\bigsqcup}\left(\overrightarrow{a},\overrightarrow{b}\right)$.
- The double digest sequence of $\overrightarrow{\mathrm{a}}$ and $\overrightarrow{\mathrm{b}}$, denoted by $\mathrm{DDS}\left(\overrightarrow{a},\overrightarrow{b}\right)$, can be defined as the step difference vector of $\hat{\bigsqcup}\left(\overrightarrow{a},\overrightarrow{b}\right)$. That is,

4 $$ \mathrm{DDS}\left(\overrightarrow{a},\overrightarrow{b}\right)= SD\left(\hat{\bigsqcup}\left(\overrightarrow{a},\overrightarrow{b}\right)\right) $$

- Now, we introduce the following definition:

#### Definition 1

*A double digest problem (DDP) instance is specified by three vectors* $\overrightarrow{a}=\left({a}_1,\cdots, {a}_m\right),\overrightarrow{b}=\left({b}_1,\cdots, {b}_n\right)\  and\;\overrightarrow{c}=\left({c}_1,\cdots, {c}_k\right)$ *with the restriction of*

5 $$ \Sigma \left(\overrightarrow{a},m\right)=\Sigma \left(\overrightarrow{b},n\right)=\Sigma \left(\overrightarrow{c},k\right) $$

*and the objective is to find a pair permutations* (*μ*, *ν*) ∈ *S*_*m*_ × *S*_*n*_ *such that.*

6 $$ DDS\left({\overrightarrow{a}}^{\mu },{\overrightarrow{b}}^v\right)={\overrightarrow{c}}^{\pi }\  for\; som\ \pi \in {S}_m $$

#### Remark 1

*If two enzymes cut a target DNA molecule at disjoint sites, then we have the condition k* = *m* + *n* − 1*. It was previously suspected that this case might lead to easier reconstruction problems* [2]*. (However, our simulation does support this conjecture, and details are given in the supplementary part). However, due to some unavoidable experimental errors, this condition does not always hold. Thus, in DDmap, we employ a very simple strategy to address the cases of k* = *m* + *n* − 1*: Introducing 0-length fragments in sequence A,B, or C if necessary. Our simulation results show that this method is considerably robust.*

#### Remark 2

*If we take into consideration possible partial cleavage errors, then the optimization goal (6) should be updated to*

7 $$ \left.{\mathit{\min}}_{\mu \in {S}_m,\nu \in {S}_n}\right|\left| DDS\left({\overrightarrow{a}}^{\mu },{\overrightarrow{b}}^{\nu}\right)\oplus \overrightarrow{c}\right| $$

*where symbol* ⊕ *indicates the set exclusive operation, and the two operands* $DDS\left({\overrightarrow{a}}^{\mu },{\overrightarrow{b}}^{\nu}\right)$ *and*$\overrightarrow{c}$ *should be regarded as unordered multisets. By doing so, the searching space of the DDP solution is reduced to S*_*m*_ × *S*_*n*_*, instead of S*_*m*_ × *S*_*n*_ × *S*_*k*_*. In fact,π can be easily extracted from any valid solution* (*μ*, *ν*)*. A simple method for obtaining π is to at first sort* $DDS\left({\overrightarrow{a}}^{\mu },{\overrightarrow{b}}^{\nu}\right)$ *to obtain a nondecreasing sequence and then let π be the permutation specified by the reverse index of the sorting subscripts. Apparently, this step can be performed within the complexity* Ο(*klogk*)*.*

#### Example 1

*For given three vectors* $\overrightarrow{a}=\left(1,2,3,5\right)$ *,* $\overrightarrow{b}=\left(2,2,3,4\right)$ *and* $\overrightarrow{c}=\left(1,1,1,2,2,2,2\right)$ *as well as two permutations.*

$$ \mu =\left(\begin{array}{c}1\ 2\ 3\ 4\\ {}2\ 4\ 3\ 1\end{array}\right) $$ *and* $$ \nu =\left(\begin{array}{c}1\ 2\ 3\ 4\\ {}3\ 1\ 2\ 4\end{array}\right) $$*, we can verify that* (*μ*, *ν*) *is a valid solution for the DDP instance specified by* $\left(\overrightarrow{a},\overrightarrow{b},\overrightarrow{c}\right)$*. The pie charts of a solution and the corresponding calculation steps and complexities are depicted in* *Table* *3**.*

**Table 3:**
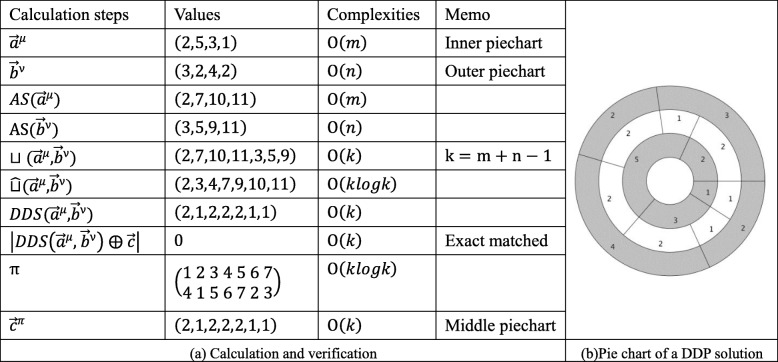
Illustration of the proposed formulation. This is the detailed process of solving the double digest problem, The calculation process of example 1 is listed in (a). The pie chart for this example’s solution is in (b).

### The proposed genetic operators

Recall that the basic idea of a genetic algorithm consists of the following concepts: an individual is totally specified by a chromosome; a chromosome is the carrier of a gene, and the position of a gene in a chromosome is called a locus; the gene composition of an individual is called the genotype; and the fitness value, called phenotype, is the result of mutual effects of genotype and external environments. Thus, to design a genetic algorithm for a given optimization problem, we need to specify how to represent a chromosome, evaluate the fitness value, design genetic operators, and determine evolution strategies such as the population size, the maximum evolution generation, the elitism keeping method, the probabilities for each genetic operator, etc.

First, for a given DDP instance $\left(\overrightarrow{a},\overrightarrow{b},\overrightarrow{c}\right)$, we directly use a random pair of permutations (*μ*, *ν*) ∈ *S*_*m*_ × *S*_*n*_ to represent a chromosome, and its fitness value is given by

8 $$ f\left(\mu, \nu \right)=\frac{1}{1+\left| DDS\left({\overrightarrow{a}}^{\mu },{\overrightarrow{b}}^{\nu}\right)\oplus \overrightarrow{c}\right|} $$

Second, the following 6 genetic operators are employed in this work:

- **RWS**. This is a natural selection operator defined as the well-known roulette wheel algorithm.
- **PCC**. This is a crossing operator defined as a combination of two permutations. Given two chromosomes (*μ*^(1)^, *ν*^(1)^) and (*μ*^(2)^, *v*^(2)^), this operator produces two new offspring

$$ \left({\mu}^{(1)}\circ {\mu}^{(2)}\circ {\nu}^{(1)}\circ {\nu}^{(2)}\right) $$

and

$$ \left({\mu}^{(2)}\circ {\mu}^{(1)}\circ {\nu}^{(2)}\circ {\nu}^{(1)}\right) $$

respectively.

- **RSC**. This is a crossing operator defined as the so-called referencing sorting (RS). Given a target sequence $\overrightarrow{a}$ and a reference sequence $\overrightarrow{b}$, assuming both are defined over the same alphabet. Then, during the sorting process, the swapping operation of two elements in $\overrightarrow{a}$ is performed only if they are in the reverse order in the referencing sequence $\overrightarrow{b}$. RS is a generalization of ordinary sorting in the sense that any two elements can be compared even if they do not come from a complete order. RS is inspired by operator precedence grammars. More details about RS and RSC are given in the supplementary section. In fact, RSC is called *order preserving weighted crossover* in [13].
- **P4X**. This is a four-point mutating operator defined as follows: Given a chromosome (*μ*, *ν*), randomly exchange two elements of μ and two elements of ν.
- **FLP**. This is a fragment mutating operator defined as flipping of the given fragment. By flipping a fragment (2, 5, 4, 1), we obtain (1, 4, 5, 2).
- **CSH**. This is a fragment mutating operator defined as cyclic shifting of the given fragment. By cyclically shifting a fragment (2, 5, 4, 1), we obtain (5, 4, 1, 2).

More details about the referenced sorting crossover (RSC) genetic operator.

RSC is in fact the order preserving weighted crossover given in [13]. Suppose two parent chromosomes are

$$ {\displaystyle \begin{array}{l}{\mathrm{p}}_1=\left(1,3,2,1,3,4,2,2\right)\mathrm{and}\\ {}{\mathrm{p}}_2=\left(1,2,2,2,4,3,3,1\right),\end{array}} $$

and the crossover point is 3. Then, the producing of the offspring is given below:

1. p_1_ is split into two pieces: p_11_ = (1, 3, 2) and p_12_ = (1, 3, 4, 2, 2), and p_2_ is split into two pieces: p_21_ = (1, 2, 2) and p_22_ = (2, 4, 3, 3, 1).
2. The piece p_12_ is sorted by taking p_2_ as the referenced sequence. Since in p_2_ there exists a chain 2 − 2 − 4 − 3 − 1 this leads to p ' _12_ = (2, 2, 4, 3, 1).
3. Similarly, p_22_ is sorted by taking p_1_ as the referenced sequence. This time, we obtain p '_ 22_ = (3, 1, 3, 4, 2) since there exists a chain 3 − 1 − 3 − 4 − 2 in p_1_.
4. Two offspring chromosomes are

$$ {\displaystyle \begin{array}{l}{\mathrm{c}}_1={\mathrm{p}}_{11}\Big\Vert {\mathrm{p}}_{12}^{\prime }=\operatorname{}\left(1,3,2,2,2,4,3,1\right)\mathrm{and}\\ {}{\mathrm{c}}_2={\mathrm{p}}_{21}\Big\Vert {\mathrm{p}}_{22}^{\prime}\operatorname{}\left(1,2,2,3,1,3,4,2\right).\end{array}} $$

Among the above 6 genetic operators, RWS is widely used in most genetic algorithms, and RSC was first used in [13] to solve the DDP problem. Four other genetic operators, although being easily conceived, are new to DDP-oriented genetic algorithms, as far as we know.

Third, the evolution strategies in this work refer to [13]. That is, the population size and maximum evolution generation are set to 50 and 10,000, respectively. Elitists in each generation are kept, and the crossing probability is set to 0.85. The linearly adaptive mutation probability in [13] is also used in our work, but with a slight modification to ensure the cyclic increment of mutation probability is nonnegative. The details are as follows:

We follow the suggestion given in [13] by letting the mutation probability vary linearly in cycles of 200 iterations. However, in the original paper, this cycle varies from $\frac{2}{m+n}$ to 0.45, while in our work, the cycle varies from $\frac{2}{m+n}$ to 0.55, considering that in the case of m = n = 2, the start point would be 0.5, which is larger than 0.45.

### Scaling-rounding-adjusting approach

Based on the above observation, we try to deal with the instance INS_2_ in another way. A new test instance, INS_2_, is derived by using a scaling-rounding-adjusting process on INS_2_. The details of this process are as follows:

- Scaling and rounding. Because the minimum length of pieces in sequence $\overrightarrow{c}$ of INS_2_ is 1120, we take 0.001 as the scaling factor. That is, we multiply the sequences $\overrightarrow{\mathrm{a}},\overrightarrow{\mathrm{b}},\overrightarrow{\mathrm{c}}$ by 0.001 and then round them. By doing so, we obtain

$$ {\displaystyle \begin{array}{c}\overrightarrow{{\mathrm{a}}^{\prime }}=\left(6,6,7,7,7,17\right)\\ {}\overrightarrow{{\mathrm{b}}^{\prime }}=\left(4,5,6,6,7,21\right)\\ {}\overrightarrow{{\mathrm{c}}^{\prime }}=\left(1,2,3,3,3,4,4,4,5,6,16\right)\end{array}} $$

- Adjusting. Next, we find that

$$ \sum \left(\overrightarrow{{\mathrm{a}}^{\prime }}\right)=50\ne \sum \left(\overrightarrow{{\mathrm{b}}^{\prime }}\right)=49\ne \sum \left(\overrightarrow{{\mathrm{c}}^{\prime }}\right)=51 $$

That is, $\left(\overrightarrow{\mathrm{a}},\overrightarrow{\mathrm{b}},\overrightarrow{\mathrm{c}}\right)$ is an invalid DDP instance. Intuitively, this occurs because the round operation, round(·), introduces more errors. Thus, we try to adjust the rounding operation in the previous step according to the so-called rounding-up and rounding-down strategies:

- Rounding-up: round(x) is replaced by x '  = round(x + 0.1), and we obtain

$$ {\displaystyle \begin{array}{c}\overrightarrow{{\mathrm{a}}^{{\prime\prime} }}=\left(6,6,7,7,7,17\right)\\ {}\overrightarrow{{\mathrm{b}}^{{\prime\prime} }}=\left(4,5,6,6,8,21\right)\\ {}\overrightarrow{{\mathrm{c}}^{{\prime\prime} }}=\left(1,2,3,3,3,4,4,4,5,6,16\right)\end{array}} $$

This DDP instance is again invalid since

$$ \sum \left(\overrightarrow{{\mathrm{a}}^{{\prime\prime} }}\right)=50=\sum \left(\overrightarrow{{\mathrm{b}}^{{\prime\prime} }}\right)\ne \sum \left(\overrightarrow{{\mathrm{c}}^{{\prime\prime} }}\right)=51 $$

- Rounding-down: round(x) is replaced by x '  = round(x − 0.1)

$$ {\displaystyle \begin{array}{c}\overrightarrow{{\mathrm{a}}^{{\prime\prime\prime} }}=\left(5,6,6,7,7,17\right)\\ {}\overrightarrow{{\mathrm{b}}^{{\prime\prime\prime} }}=\left(3,5,6,6,7,21\right)\\ {}\overrightarrow{{\mathrm{c}}^{{\prime\prime\prime} }}=\left(1,2,2,3,3,3,4,4,5,5,16\right)\end{array}} $$

Now, the DDP instance may be valid since

$$ \sum \left(\overrightarrow{{\mathrm{a}}^{{\prime\prime\prime} }}\right)=\sum \left(\overrightarrow{{\mathrm{b}}^{{\prime\prime\prime} }}\right)=\sum \left(\overrightarrow{{\mathrm{c}}^{{\prime\prime\prime} }}\right)=48 $$

Note that the constant 0.1 in rounding-up/rounding-down is a value defined by experience. A reasonable domain of this constant would be the interval [0.0001, 0.4999].

• Now, the newly derived DDP instance INS_2_, is given by the three vectors $\left(\overrightarrow{{\mathrm{a}}^{{\prime\prime\prime} }},\overrightarrow{{\mathrm{b}}^{{\prime\prime\prime} }},\overrightarrow{{\mathrm{c}}^{{\prime\prime\prime} }}\right)$.

Finally, we would like to mention that all simulations in this work are conducted on a X1 Carbon laptop with Windows(TM) 8, Intel(R)Core(TM)i5 − 4300U CPU @ 1.90 GHz/2.49 GHz and 8GB RAM. The complete genetic algorithm for solving the DDP problem is implemented as a MATLAB package, DDmap, and a Maple source code for drawing DDP solutions as nested pie charts is also included in this package.

The package DDmap consists of

- 13 MATLAB algorithms:
- permGA.m, the MATLAB genetic algorithm for solving the DDP problem. This is the main algorithm, and its flowchart is depicted in Fig. 7. Note that this file also contains the definitions of five genetic operators — RWS, PCC, P4X, FLP, CSH and related MATLAB functions for calculating the fitness values.

- referIndexSort.m, the MATLAB algorithm for implementing the so-called referenced sorting (based on index).
- opPermCross.m, the MATLAB algorithm for implementing the RSC genetic operator.
- getInstance.m, the auxiliary MATLAB algorithm for outputting test DDP instances in [Sur-Kolay S. et al., 2005].
- randDDPinstance.m, the auxiliary MATLAB algorithm for producing a valid DDP instance according the given parameters.
- strABC.m, the auxiliary MATLAB algorithm for producing Maple commands for reading data before calling the Maple algorithm DDdraw.mws.
- simu1004.m, simu1004plots.m, simu1007.m, and simu1008.m, the auxiliary MATLAB algorithms for organizing simulations and producing the related figures.
- Trans.m, the MATLAB algorithm for implementing Scaling-rounding-adjusting approach for Cases of INS_2._
- Plot1.m, the auxiliary MATLAB algorithms for comparison of DDmap and algorithm in [3, 13].
- Plot2.m, the auxiliary MATLAB algorithms for comparison of DDmap and other algorithms under the condition of INS_2._
- 1 Maple algorithm, DDdraw.mws, is used for drawing the DDP solution in nested pie charts, with inputs A, B and C that are assigned by using Maple commands produced by strABC.m.
- 43 Data files: 42 of them are named as INS_xx − ggg_. TEX, where xx ∈ {01, 03, 04, 05, 07, 08, 10} and ggg ∈ {pcc, rsc, p4x, flp, csh, all}, and the last is named as INS_02 − rs − 1008_. TEX. These data files are in fact the running records of our simulations towards the 7 valid DDP test instances given in [13]. In these running records, many exact DDP solutions are provided.

## Abbreviations

CSH: Cyclic shifting
DDE: Double digest experiments
DDP: Double digest problem
FLP: Flipping
P4X: Four-point exchange
PCC: Permutations combination crossing
RSC: Referencing sorting crossing
RWS: Roulette wheel selection
